# Migration of silicone oil into the ventricular system, a rare neurological complication

**DOI:** 10.1055/s-0046-1818607

**Published:** 2026-04-07

**Authors:** Karina Contin Carvalho, Bernardo Carvalho Muniz

**Affiliations:** 1 Hospital Alcides Carneiro, Departamento de Radiologia, Petrópolis RJ, Brazil.; 2 Instituto Estadual do Cérebro Paulo Niemeyer, Departamento de Radiologia, Rio de Janeiro RJ, Brazil.

**Figure 1:**
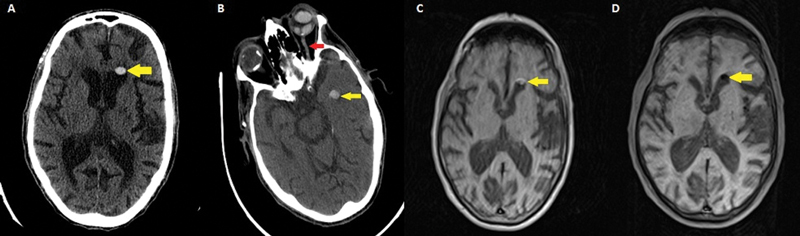
Intraventricular migration of silicone oil after vitrectomy. ( **A** ) Axial computed tomography (CT) scan, brain parenchyma window, showing a hyperdense, rounded, oval-shaped image within the frontal horn of the left lateral ventricle. ( **B** ) Axial CT scan with the head tilted and brain parenchyma window, showing migration of the lesion into the left temporal horn of the corresponding lateral ventricle (yellow arrow), indicating lesion mobility without morphological changes. There is also hyperdensity of the left optic nerve throughout its entire length (red arrow) and diffuse hyperdensity of the left eyeball due to vitrectomy using silicone oil. ( **C** , **D** ) Magnetic resonance imaging scan of the brain, in the axial plane, with anatomical head position, in T1-weighted images without and with fat saturation respectively, showing the lesion with high signal intensity on the T1-weighted images, with a decrease in signal intensity after fat saturation, confirming the fatty content of the lesion.

We herein report the case of a 75-year-old male patient with a history of vitrectomy and silicone oil tamponade in the left eye, with moderate holocranial headache and disorientation. The physical examination showed no focal neurological deficits, and the vital signs were normal. A cranial computed tomography (CT) scan revealed a hyperdense, oval image in the left lateral ventricle, initially suggestive of intraventricular hemorrhage ( Figure 1 ). A follow-up CT scan, acquired in an oblique position, showed the image had migrated to the frontal horn of the left lateral ventricle. The diagnosis of intraventricular silicone-oil migration was established, representing a late vitrectomy complication. A magnetic resonance imaging (MRI) scan confirmed lipid content within the lesion. 1 2
